# A Model for Community‐based Recruitment for Dementia Clinical Trials

**DOI:** 10.1002/alz70859_106584

**Published:** 2025-12-25

**Authors:** Brittany Prokop, Natalie Dren, Winnie Qian, Victoria Telford, Jordanne Holland, Tarek K Rajji, David F. Tang‐Wai, Luca F Pisterzi

**Affiliations:** ^1^ Toronto Dementia Research Alliance, Toronto, ON Canada; ^2^ Baycrest Hospital, Toronto, ON Canada; ^3^ Centre for Addiction and Mental Health, Toronto, ON Canada; ^4^ Department of Psychiatry, Temerty Faculty of Medicine, University of Toronto, Toronto, ON, Canada, Toronto, ON Canada; ^5^ UT Southwestern University, Dallas, TX USA; ^6^ Memory Clinic, Toronto Western Hospital, University Health Network, Toronto, ON Canada; ^7^ Temerty Faculty of Medicine, University of Toronto, Toronto, ON Canada; ^8^ Alzheimer Society of Canada, Toronto, ON Canada

## Abstract

**Background:**

Successful enrollment of participants into clinical dementia trials remains one of the more challenging aspects of clinical research, as barriers exist for both potential participants and recruiting clinicians. Currently, a mechanism is lacking to engage the community.

**Method:**

The Toronto Dementia Research Alliance (TDRA) and the Alzheimer Society of Toronto (AST) co‐developed a web recruitment platform, Toronto Dementia Network (TDN) (https://tdn.alz.to/research‐studies/) to enable persons living with dementia, their caregivers, and healthy volunteers, to discover trials and to connect clinicians to otherwise inaccessible participants. The platform features plain‐language summaries of investigator‐initiated clinical trials. Potential participants can perform key‐word searches, use predetermined filters, or fill out a questionnaire to be matched to a suitable trial by TDRA staff. Their contact information is securely captured and shared with the site leading the trial of interest. Once transferred, staff leading that trial contact the individual who made the request. This triage system is tracked and monitored by TDRA. This platform is featured in TDRA’s monthly newsletter and through a bi‐monthly webinar series.

**Result:**

From May 2021 to January 30^th^ 2025, 60 studies have been registered on the TDN site. 266 potential participants have demonstrated interest. Overall, this method has led to 34% of participant referrals becoming enrolled in a study. Specifically, 36% of referrals via TDRA‐assisted screening and study matching have led to enrollments/study completions, while 30% of participant self‐referrals have led to enrollments/study completions – higher than traditional recruitment methods.

**Conclusion:**

A patient‐friendly web‐based approach for participation into clinical trials is possible. We have demonstrated that such an approach can yield over 30% recruitment. In addition, having a research team dedicated to screening, triaging and matching participants to appropriate studies results in a higher enrollment rate than self‐referrals.

